# Nutrition and the Gut Microbiota in 10- to 18-Month-Old Children Living in Urban Slums of Mumbai, India

**DOI:** 10.1128/mSphere.00731-20

**Published:** 2020-09-23

**Authors:** Samantha L. Huey, Lingjing Jiang, Marcus W. Fedarko, Daniel McDonald, Cameron Martino, Farhana Ali, David G. Russell, Shobha A. Udipi, Aparna Thorat, Varsha Thakker, Padmini Ghugre, R. D. Potdar, Harsha Chopra, Kripa Rajagopalan, Jere D. Haas, Julia L. Finkelstein, Rob Knight, Saurabh Mehta

**Affiliations:** a Division of Nutritional Sciences, Cornell University, Ithaca, New York, USA; b Department of Bioengineering, University of California, San Diego, California, USA; c Center for Microbiome Innovation, University of California San Diego, La Jolla, California, USA; d Department of Computer Science and Engineering, University of California San Diego, La Jolla, California, USA; e Department of Pediatrics, School of Medicine, University of California San Diego, La Jolla, California, USA; f Bioinformatics and Systems Biology Program, University of California San Diego, La Jolla, California, USA; g Department of Microbiology and Immunology, Cornell University, Ithaca, New York, USA; h Department of Nutrition and Food Science, SNDT Women’s University, Mumbai, India; i Centre for the Study of Social Change, Mumbai, India; j Institute for Nutritional Sciences, Global Health, and Technology (INSiGHT), Cornell University, Ithaca, New York, USA; k Division of Biostatistics, University of California, San Diego, California, USA; University of Michigan—Ann Arbor

**Keywords:** infant, child, growth, diet, nutrition, feeding practices, microbiome, fat intake, anthropometry

## Abstract

In this cross-sectional study, we describe the composition and diversity of the gut microbiota among undernourished children living in urban slums of Mumbai, India, and determine how nutritional status, including anthropometric measurements, dietary intakes from complementary foods, feeding practices, and micronutrient concentrations, is associated with their gut microbiota. We collected rectal swabs from children aged 10 to 18 months living in urban slums of Mumbai participating in a randomized controlled feeding trial and conducted 16S rRNA sequencing to determine the composition of the gut microbiota. Across the study cohort, *Proteobacteria* dominated the gut microbiota at over 80% relative abundance, with *Actinobacteria* representation at <4%, suggesting immaturity of the gut. Increased microbial α-diversity was associated with current breastfeeding, greater head circumference, higher fat intake, and lower hemoglobin concentration and weight-for-length Z-score. In redundancy analyses, 47% of the variation in Faith’s phylogenetic diversity (Faith’s PD) could be accounted for by age and by iron and polyunsaturated fatty acid intakes. Differences in community structure (β-diversity) of the microbiota were observed among those consuming fats and oils the previous day compared to those not consuming fats and oils the previous day. Our findings suggest that growth, diet, and feeding practices are associated with gut microbiota metrics in undernourished children, whose gut microbiota were comprised mainly of *Proteobacteria*, a phylum containing many potentially pathogenic taxa.

**IMPORTANCE** The impact of comprehensive nutritional status, defined as growth, nutritional blood biomarkers, dietary intakes, and feeding practices, on the gut microbiome in children living in low-resource settings has remained underreported in microbiome research. Among undernourished children living in urban slums of Mumbai, India, we observed a high relative abundance of *Proteobacteria*, a phylum including many potentially pathogenic species similar to the composition in preterm infants, suggesting immaturity of the gut, or potentially a high inflammatory burden. We found head circumference, fat and iron intake, and current breastfeeding were positively associated with microbial diversity, while hemoglobin and weight for length were associated with lower diversity. Findings suggest that examining comprehensive nutrition is critical to gain more understanding of how nutrition and the gut microbiota are linked, particularly in vulnerable populations such as children in urban slum settings.

## Abstract (precis)

The impact of comprehensive nutritional status, defined as growth, nutritional blood biomarkers, dietary intakes, and feeding practices, on the gut microbiome in children living in low-resource settings has remained underreported in microbiome research. Among undernourished children living in urban slums of Mumbai, India, we observed a high relative abundance of *Proteobacteria*, a phylum including many potentially pathogenic species similar to the composition in preterm infants, suggesting immaturity of the gut, or potentially a high inflammatory burden. We found head circumference, fat and iron intake, and current breastfeeding were positively associated with microbial diversity, while hemoglobin and weight for length were associated with lower diversity. Findings suggest that examining comprehensive nutrition is critical to gain more understanding of how nutrition and the gut microbiota are linked, particularly in vulnerable populations such as children in urban slum settings.

## INTRODUCTION

The dynamics of the developing gut microbiota in later infancy and early childhood have not been fully explored, compared to early infancy, and particularly in low-resource settings. Perturbations in the microbiota of infants and young children have been associated with many factors, including nutrition status, diet, delivery mode, infections, and immune function, including antibiotic or other medication administration (1). It is accepted that the infant gut microbiota are first represented by facultative anaerobes in the *Proteobacteria* phylum and succeed in favor of *Actinobacteria* and in particular, *Bifidobacteria* spp. with the introduction of breast milk (2). By the time the child is 2 to 3 years of age, the microbiota in early life reach an adult-like balance mainly consisting of *Firmicutes* and *Bacteroidetes* phyla with depleted *Proteobacteria* and even lower *Actinobacteria* (2). However, the factors associated with this transition are less understood.

Diet plays a direct role in the gut microbiota composition and function (3). Food components indigestible by humans (such as fiber [4]) are broken down by commensal microbiota in the gut to later serve as signaling molecules such as short-chain fatty acids (SCFAs) for other human cells such as immune cells as well as nutrients for commensal bacteria (5, 6). In infants, both indirect and direct dietary intakes are thought to be a major driver of gut composition, from maternal diet during pregnancy and lactation (7), to consumption by infants. Breast milk and formula have also been shown to be associated with differences in the composition of the gut microbiota; for example, breastfed infants tend to have lower bacterial diversity (8) and a higher relative abundance of beneficial *Bifidobacterium* species, than formula-fed infants who tend to have higher abundances of Escherichia coli and Clostridium difficile (9). However, few studies have examined changes in the gut microbiota during weaning, with the introduction to complementary foods.

It is well established that immune function and health outcomes differ by diet and nutritional status during the first few years of life, particularly in low-resource settings (10). Ascertaining differences in the gut microbiota by nutritional status and dietary intake may represent a mediating factor in immune functional capacity, as gut microbiota and immune function develop in tandem (1), involving commensal microbiota-host immune cell cross talk, signaling, and education (11, 12).

Most studies examining early life development of the gut microbiota have been performed in higher-income settings (13–29) or in low-income settings such as Bangladesh (30) and Malawi (31). However, an analysis of the gut microbiota during the second year of life in young children living in a low-resource urban slum setting of Mumbai, India—where poor growth (as measured by anthropometric length-for-age Z-score [LAZ], weight-for-age Z-score [WAZ], and weight-for-length Z-score [WLZ] [32]), infections, and poor sanitation are common (33–35)—has yet to be described. Therefore, the objective of this cross-sectional analysis was to (i) characterize the gut microbiota among 10- to 18-month-old children in Mumbai’s urban slums and (ii) determine the association between comprehensive nutritional status (as determined by anthropometric measurements, blood nutritional biomarkers, dietary intakes, and feeding practices) and relative abundance, α-diversity, and β-diversity of the gut microbiota.

## RESULTS

### Demographic characteristics.

Participant characteristics of the samples (*n* = 53) are described in Table 1. Children were sampled from five urban slum communities in Mumbai, India. Nearly 30% of children were stunted (LAZ < −2), 25% underweight (WAZ < −2), and 12% wasted (WLZ < −2). From a subset of participants with hemoglobin data, 77% were anemic, and from a subset of participants with nutritional blood biomarker data, 74% were iron deficient. Most children consumed a nonvegetarian diet and were reported to be currently breastfeeding. Comparison of characteristics between the included cohort in this report (*n* = 53) and the rest of the screened population (*n* = 312) from the parent trial is in Table S1 in the supplemental material. Participants did not differ in most characteristics; however, more children were born by Caesarean section (compared to vaginal birth) in the current study.

**TABLE 1:** Participant characteristics

Parameter	*n* ^*a*^	Median (IQR) or *n* (%)
Sociodemographic		
Age (mo)	53	14.8 (13.1, 16.7)
Female	53	25 (47.2)
Vaginally delivered (versus Caesarean)	51	29 (56.9)
Anthropometry		
Birth weight (kg)	53	2.7 (2.5, 3.0)
Low birth weight (<2.5 kg)	53	10 (18.9)
Current weight (kg)	53	8.7 (8.0, 9.8)
Mid-upper arm circumference (cm)	53	14.8 (14.1, 15.1)
Head circumference (cm)	53	44.2 (43.0, 45.4)
Head circumference-for-age Z-score	52	−1.58 (−2.12, −0.73)
Length (cm)	51	74.0 (72.1, 77.7)
Length-for-age Z-score (LAZ)	51	−1.29 (−2.38, −0.46)
Stunting	51	15 (29.4)
Weight-for-age Z-score (WAZ)	53	−0.96 (−1.99, −0.34)
Underweight	53	13 (24.5)
Weight-for-length Z-score (WLZ)	51	−0.68 (−1.34, 0.08)
Wasting	51	6 (11.8)
Blood biomarkers and illness history		
Ferritin (ng/ml)	44	7.55 (3.20, 15.70)
Iron deficiency (<12 ng/ml)	44	27 (61.4)
Zinc (μmol/liter)	38	13.18 (11.08, 15.28)
Zinc deficiency (<9 μmol/liter)	38	0 (0)
Hemoglobin (g/dl)	43	10.10 (9.10, 10.80)
Anemia (hemoglobin < 11 g/dl)	43	33 (76.7)
C-reactive protein (CRP) ≥ 5 mg/liter	39	3 (7.7)
Diarrhea today or within past 4 weeks	51	9 (17.7)
Fever today or within past 4 weeks	51	19 (37.3)
Cough today or within past 4 weeks	51	7 (13.7)
Dietary intakes^*b*^		
Calories (kcal)	52	393.0 (270.0, 645.5)
Protein (g)	52	13.6 (8.1, 21.6)
Fat (g)	52	12.9 (8.9, 20.8)
Saturated fat (g)	52	4.5 (0.8, 5.8)
Monounsaturated fat (g)	52	2.2 (0.4, 3.0)
Polyunsaturated fat (g)	52	0.4 (0.3, 0.7)
Carbohydrate (g)	52	65.3 (33.6, 85.9)
Fiber (g)	52	2.0 (0.5, 5.5)
Calcium (mg)	52	216.5 (62.0, 349.5)
Iron (mg)	52	2.0 (1.2, 3.3)
Zinc (mg)	52	1.3 (0.8, 2.0)
Vitamin A (μg RAE)	52	99.0 (17.0, 141.0)
Cobalamin (vitamin B_12_) (μg)	52	0 (0, 0)
Feeding practices		
Diet: Vegetarian (including eggs)	51	7 (13.7)
Diet: Vegetarian (no eggs)	51	15 (29.4)
Diet: Nonvegetarian	51	29 (56.9)
Ever breastfed	52	48 (92.3)
Breastfed yesterday (current breastfeeding)	48	40 (83.3)
Exclusive breastfeeding duration (months)	51	7.0 (6.0, 7.0)
Exclusively breastfed > 6 months	51	33 (64.7)
Consumed grains (bread, rice, noodles, porridge) yesterday^*c*^	51	47 (92.2)
Consumed any fruits and vegetables yesterday^*d*^	51	23 (45.1)
Liver, kidney, heart, or other organ meats^*e*^	50	2 (4.0)
Consumed beef, pork, poultry yesterday^*f*^	51	7 (13.7)
Consumed eggs yesterday^*g*^	51	10 (19.6)
Consumed dried fish or seafood yesterday^*h*^	51	2 (3.9)
Consumed beans, peas, lentils, nuts, or seeds yesterday^*i*^	51	22 (43.1)
Consumed dairy yesterday^*j*^	50	26 (52.0)
Consumed oil or butter yesterday^*k*^	51	13 (25.5)
Consumed sugary foods yesterday^*l*^	51	30 (58.8)
Consumed condiments yesterday^*m*^	50	6 (12.0)

a *n* is the number of participants in the study.

b Dietary intakes as absolute (unadjusted) values.

c Infant and Young Child Feeding (IYCF) (World Health Organization) food group A.

d IYCF food groups B to F combined (orange and starchy root vegetables, dark leafy green vegetables, ripe mangoes or papayas, any other fruits and vegetables).

e IYCF food group G.

f IYCF food group H.

g IYCF food group I.

h IYCF food group J.

i IYCF food group K.

j IYCF food group L.

k IYCF food group M.

l IYCF food group N.

m IYCF food group O.

10.1128/mSphere.00731-20.3 TABLE S1 Comparison of microbiome subset with excluded screening population. Download Table S1, DOCX file, 0.02 MB. Copyright © 2020 Huey et al. 2020 Huey et al. This content is distributed under the terms of the Creative Commons Attribution 4.0 International license.

### Gut microbiota 16S sequencing.

From 53 participants, a total of 16,778,710 forward and reverse reads were processed. After initial quality filtering and trimming (see Materials and Methods for more details) (36), the number of joined paired-end reads totaled 7,554,901, with 9,251 unique sequences across the study population (median 138,928 [interquartile range {IQR}, 94,704, 175,232] reads per sample). Filtering out mitochondria and chloroplasts removed 18,119 sequences for a total frequency of 7,536,782 sequence variants, including 2,248 unique sequences (median 138,834 {IQR, 94,357, 174,909]) per sample, with a minimum sequence frequency per sample of 38,012.

### Relative abundance.

**FIG 1:**
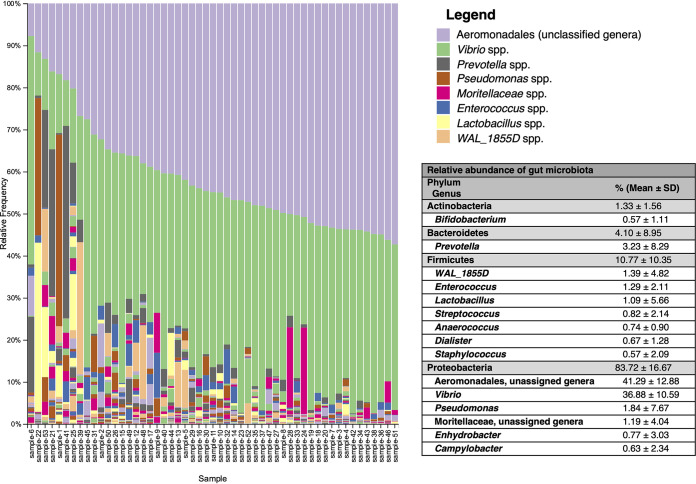
Relative abundance of genera across participants. Each stacked bar plot corresponds to one infant subject. Figure legend colors repeat for additional identified taxa. Please see Fig. S1B in the supplemental material for full legend.

Rarefaction to 38,012 reads per sample retained 2,014,636 sequences (26.73% of total sequences). Genera of the phylum *Proteobacteria* (Fig. 1; see also Fig. S1 in the supplemental material) dominantly represented the gut microbiota. Approximately 128 identified and unidentified genera were found across the population. The *Aeromonadales* order (unspecified genera) and genus *Vibrio* spp. combined represented over 75% of all sequences across all participants (Fig. 1). Additional genera represented at greater abundance (>1%) included *Prevotella*, *Pseudomonas*, and *Enterococcus*, with genera at minimal representation (mean abundance of <1%) including *Streptococcus*, *Enhydrobacter*, *Anaerococcus*, *Dialister*, *Campylobacter*, *Bifidobacterium*, and *Staphylococcus* (Fig. 1). A full legend of all genera may be found in Fig. S1.

10.1128/mSphere.00731-20.1 FIG S1 Full legend for gut microbiota relative abundance of phyla (A) and genera (B). Figure legend colors repeat for additional identified taxa. Download FIG S1, TIF file, 2.3 MB. Copyright © 2020 Huey et al. 2020 Huey et al. This content is distributed under the terms of the Creative Commons Attribution 4.0 International license.

After correction for multiple comparisons using the Benjamini-Hochberg false discovery rate (FDR) (37), multivariate linear regressions showed no nutritional exposures associated with percent relative abundance of the four main phyla present in gut microbiota: *Proteobacteria*, *Firmicutes*, *Bacteroidetes*, or *Actinobacteria* (Table 2). All linear regressions examining associations between nutritional exposures and phylum relative abundance are included in Table S2.

**TABLE 2:** Correlates of phylum relative abundance in multivariate analyses^*a*^

Taxon and parameter	*n*	β (%) (95% CI)	*P* value^*b*^	*P* value adj.^*c*^
*Actinobacteria* (%)				
Head circumference (cm)	52	0.30 (0.02, 0.57)	**0.0327**	0.0719
Age (mo)	52	0.14 (−0.05, 0.33)	0.1439	0.1759
Female	52	−0.39 (−1.23, 0.45)	0.3660	0.4026
*Bacteroidetes* (%)				
Serum zinc (μmol/liter)	38	1.41 (0.21, 2.61)	**0.0211**	0.0719
Age (mo)	38	0.51 (−0.83, 1.85)	0.4570	0.4570
Female	38	4.63 (−1.40, 10.66)	0.1320	0.1759
*Firmicutes* (%)				
Iron (mg)^*d*^	48	7.50 (1.98, 13.03)	**0.0078**	0.0572
Log energy (kcal)^*d*^	48	−3.81 (−8.27, 0.65)	0.0944	0.1731
Age (mo)	48	1.34 (0.16, 2.53)	**0.0264**	0.0719
Female	48	4.05 (−1.17, 9.27)	0.1280	0.1759
*Proteobacteria* (%)				
Weight-for-length Z-score (WLZ)^*e*^	51	5.33 (1.25, 9.41)	**0.0104**	0.0572

a Analyses were performed using complete case analysis; similar results were found using missing indicators with median imputation (not shown).

b Boldface *P* values are statistically significant (*P* < 0.05).

c *P* value adj., adjusted *P* value corrected for multiple comparisons using the Benjamini-Hochberg false discovery rate (FDR).

d Iron intake residual adjusted for energy.

e Not adjusted for age and sex, as calculation of WLZ incorporates age and sex.

10.1128/mSphere.00731-20.4 TABLE S2 Correlates of phylum relative abundance by all tests. Download Table S2, DOCX file, 0.02 MB. Copyright © 2020 Huey et al. 2020 Huey et al. This content is distributed under the terms of the Creative Commons Attribution 4.0 International license.

### α-Diversity.

Across the study population, α-diversity metrics included Shannon diversity index (SDI), a measure of taxon diversity and evenness (38), and Faith’s phylogenetic diversity (Faith’s PD), which accounts for the phylogenetic distance between taxa within each sample (39). The median (IQR) SDI and Faith’s PD were 3.77 (3.39, 4.33) and 13.41 (11.15, 15.32), respectively. In multivariate linear regression, greater head circumference was positively associated with a 0.23 (95% confidence interval [95% CI], 0.09, 0.37) unit increase in SDI, while higher weight-for-length Z-score was associated with a 0.31 (95% CI, 0.13, 0.49) unit decrease in SDI (Table 3). In sex-stratified analysis, WLZ remained inversely associated with SDI only in male children (−0.35 [95% CI, −0.57, −0.13]; *P* = 0.002), while head circumference remained positively associated with SDI only in female children (0.28 [95% CI, 0.16, 0.40]; *P* < 0.0001).

**TABLE 3:** Correlates of α-diversity in multivariate analyses^*a*^

Correlate and parameter	*n*	β (95% CI)	*P* value^*b*^	*P* value adj.^*c*^
Shannon diversity index (SDI)				
Head circumference (cm)	51	0.21 (0.08, 0.33)	**0.001**	**0.0023**
Weight-for-length Z-score (WLZ)	51	−0.31 (−0.49, −0.13)	**0.0007**	**0.0021**
Faith’s phylogenetic diversity (PD)				
Head circumference (cm)	37	0.77 (0.34, 1.20)	**0.0004**	**0.0018**
Hemoglobin (g/dl)	37	−0.58 (−1.06, −0.10)	**0.0183**	**0.0206**
Fat (g)^*d*^	37	2.91 (1.33, 4.48)	**0.0003**	**0.0018**
Log energy (kcal)^*e*^	37	−1.58 (−2.67, −0.49)	**0.0045**	**0.0081**
Breastfed yesterday (current breastfeeding)	37	2.27 (0.53, 4.02)	**0.0106**	**0.0153**
Age (mo)	37	0.31 (0.03, 0.60)	**0.0298**	**0.0298**
Female	37	−1.58 (−2.81, −0.35)	**0.0119**	**0.0153**

a Analyses were performed using complete case analysis; similar results were found using missing indicators with median imputation (not shown).

b Boldface *P* values are statistically significant (*P* < 0.05).

c *P* value adj., adjusted *P* value corrected for multiple comparisons using the Benjamini-Hochberg false discovery rate (FDR).

d Not adjusted for age and sex, as calculation of WLZ incorporates age and sex.

e Fat residual adjusted for energy.

Head circumference was also associated with a 0.77 (95% CI, 0.37, 1.20) unit increase in Faith’s PD. Other nutritional exposures positively associated with Faith’s PD included fat intake, current breastfeeding, and child’s age in multivariate regression, while increasing hemoglobin concentration was inversely associated with Faith’s PD, and female children had lower Faith’s PD than male children. All associations remained significant after correction for multiple comparisons (Table 3), and all linear regressions examining α-diversity as an outcome are shown in Table S3.

10.1128/mSphere.00731-20.5 TABLE S3 Correlates of α-diversity by all tests. Download Table S3, DOCX file, 0.02 MB. Copyright © 2020 Huey et al. 2020 Huey et al. This content is distributed under the terms of the Creative Commons Attribution 4.0 International license.

**FIG 2:**
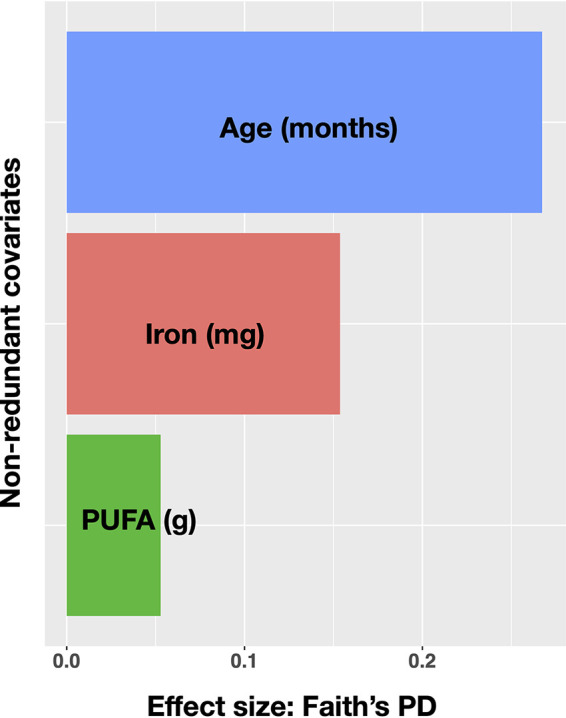
Redundancy analysis (RDA) for Faith’s phylogenetic diversity (Faith’s PD) of sociodemographic, clinical, feeding practices, and dietary correlates (dietary intakes are nutrient residuals adjusted for energy after removing colinear variables). Factors are sorted according to their effect size in the sample population and colored for distinguishability. PUFA, polyunsaturated fatty acids.

Redundancy analysis (RDA) of sociodemographic, clinical, dietary intakes as nutrient residuals adjusted for energy, and feeding practices revealed that after removing colinear variables, the age of the child had the largest explanatory power (27%) on variation in Faith’s PD, followed by iron intake (15%) and intake of polyunsaturated fatty acids (5%) (Fig. 2; details may be found in Table S4). No correlates were associated with SDI in redundancy analysis.

10.1128/mSphere.00731-20.6 TABLE S4 Redundancy analysis for α-diversity. Download Table S4, DOCX file, 0.01 MB. Copyright © 2020 Huey et al. 2020 Huey et al. This content is distributed under the terms of the Creative Commons Attribution 4.0 International license.

### β-Diversity.

**FIG 3:**
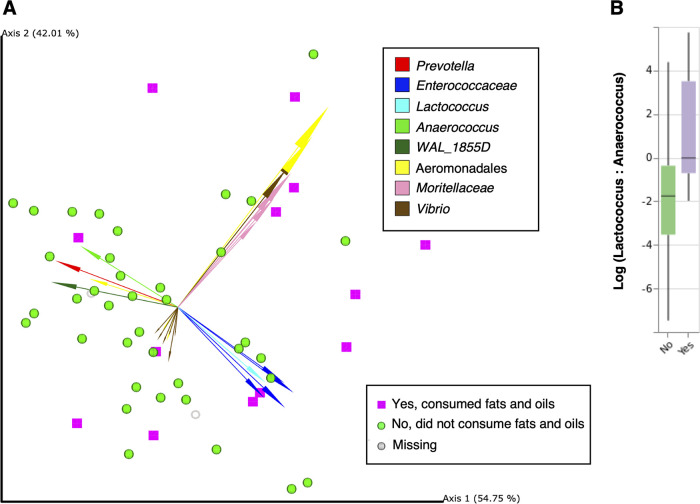
(A) DEICODE biplot showing distances among samples from children consuming fats and oils the previous day (IYCF food group M) (purple squares) compared to samples from children not consuming fats and oils the previous day (green spheres) (PERMANOVA, *P* = 0.04). Samples with missing data (*n* =  2) are represented by gray rings. (B) *Lactococcus*/*Anaerococcus* log ratio between groups reporting “yes” versus “no” showing the 37 (69.81%) samples containing a valid log ratio (not containing zero). The difference in the *Lactococcus*/*Anaerococcus* log ratio was statistically significant (*P* = 0.01).

Differences in community structure between groups were measured using unweighted and weighted UniFrac distances and tested for significant differences by permutational multivariate analysis of variance (PERMANOVA) (40) as well as PERMDISP (41) to test for dispersion, in addition to DEICODE robust Aitchison distances (42) and Qurro (43) to determine specific taxa driving clustering (Table S5). No nutritional exposures were associated with differences in community structures measured by either unweighted or weighted UniFrac distances (Table 4). Robust Aitchison distances produced by DEICODE assessed by PERMANOVA were significantly different between samples from subjects who did and did not consume oils and fats the previous day (Infant and Young Child Feeding [IYCF] food group M; Fig. 3A, *P* = 0.04) (Table 4; Table S6). The specially separated arrows in the compositional biplot revealed ratios of features classified of the phylum *Firmicutes* (lowest classified taxonomic level; *Enterococcaceae*, *Enterococcus*, *Lactococcus*, *Anaerococcus*, *WAL_1855D*) and *Proteobacteria* (lowest classified taxonomic level; *Aeromonadales*, *Moritellaceae*, *Vibrio*) that drove sample separation (Fig. 3A; Fig. S2A; see Table S5 for full list of features). By inspecting the biplot, we selected features spatially separating oil and fat consumption and visualized the corresponding log ratios using Qurro, which were assessed by *t* tests (Fig. 3A). We repeated this for 17 separate log ratios according to the cluster in which they were identified. We found that the log ratio of *Lactococcus* to *Anaerococcus* was significantly higher in the group consuming oils and fats (median [IQR], 2.62 [1.05, 6.15] versus 1.05 [−2.25, 3.57]; *P* = 0.01) (Fig. 3B). Figure S2B shows the Qurro rank plot with the *Lactococcus*/*Anaerococcus* log ratio highlighted.

**TABLE 4:** Correlates of β-diversity

Characteristic	PERMANOVA^*a*^						PERMDISP^*b*^						PERMANOVA		
	Unweighted UniFrac			Weighted UniFrac			Unweighted UniFrac			Weighted UniFrac			DEICODE^*c*^		
	Test statistic	*P* value	q- value^*d*^	Test statistic	*P* value	q- value	Test statistic	*P* value	q- value	Test statistic	*P* value	q- value	Test statistic	*P* value	q- value
Consumed oil or butter yesterday^*e*^	1.39	0.05	0.04	0.81	0.45	0.44	0.19	0.75	0.77	0.53	0.50	0.47	3.54	**0.04**	**0.04**

a PERMANOVA, permutational multivariate analysis of variance.

b PERMDISP, test for homogeneity of multivariate dispersions.

c DEICODE, robust Aitchison principal component analysis (RPCA) to determine which taxa strongly influence clustering. A boldface *P* value or q-value is statistically significant (*P* < 0.05).

d q-values derived from pairwise testing and represent the false discovery rate (FDR) analog of a *P* value.

e Infant and Young Child Feeding (IYCF) (World Health Organization) food group M.

10.1128/mSphere.00731-20.2 FIG S2 (A) DEICODE biplot shown in Fig. 3, distinguishing all identified taxa, including 2 taxa with species-level classifications (which are likely unreliable due to the resolution inherent to 16S rRNA sequencing). (B) Rank plot of *Lactococcus* to *Anaerococcus* (Qurro). Middle portion trimmed after exporting the figure from Qurro. Download FIG S2, TIFF file, 0.5 MB. Copyright © 2020 Huey et al. 2020 Huey et al. This content is distributed under the terms of the Creative Commons Attribution 4.0 International license.

10.1128/mSphere.00731-20.7 TABLE S5 Twenty-five highest-magnitude features in the DEICODE biplot. Download Table S5, DOCX file, 0.02 MB. Copyright © 2020 Huey et al. 2020 Huey et al. This content is distributed under the terms of the Creative Commons Attribution 4.0 International license.

10.1128/mSphere.00731-20.8 TABLE S6 Correlates of β-diversity by all tests. Download Table S6, DOCX file, 0.02 MB. Copyright © 2020 Huey et al. 2020 Huey et al. This content is distributed under the terms of the Creative Commons Attribution 4.0 International license.

## DISCUSSION

In this cross-sectional study, we examined the gut microbiota of 10- to 18-month-old children living in urban slums of Mumbai, India, and determined associations with comprehensive nutritional status. Overall, children were undernourished with high proportions of poor growth and nutrient deficiency, with the *Proteobacteria* phylum representing the majority of taxa in their gut microbiota. Multivariate analyses revealed differences in gut microbiota composition and measures of diversity in association with nutritional markers, including anthropometry indicators such as head circumference and weight for length; blood nutritional biomarkers, including hemoglobin; dietary fat and iron intakes; and feeding practices, such as current breastfeeding and consumption of fats and oils. Taxa from the *Firmicutes* and *Proteobacteria* phyla were identified as driving the gut microbiome sample separation globally in this cohort. Among these phyla, species in the *Enterococcus* genus, *Anaerococcus* genus, *Aeromonadales* order, and *Vibrio* genus were of high relative abundance; these have been previously shown to cause infection and harbor antibiotic resistance (44).

### Dominance of *Proteobacteria*.

Members of the *Proteobacteria* phylum dominated the gut microbiota among infants and children in this study, contrary to our expectation that *Bifidobacteria* would be in abundance due to the report of current breastfeeding in over 80% of participants. A high abundance of *Proteobacteria* has been considered a “marker for dysbiosis” or gut microbial imbalance and associated with negative health outcomes (45). For example, preterm newborns tend to have greater *Proteobacteria* abundance in their gut microbiota compared to full-term newborns (46–48), which has been shown to be associated with necrotizing enterocolitis (NEC), a devastating and potentially fatal disease in which the underdeveloped intestinal wall is invaded by bacteria with subsequent inflammation (49). In particular, higher abundance of the *Gammaproteobacteria* class has been observed in premature infants with NEC (50); interestingly, we found that most of the bacterial sequences in the data set in urban slums of Mumbai, India, were classified as *Gammaproteobacteria*. Previous studies have also found associations between *Proteobacteria* abundance and poor health states in other age groups, such as inflammatory bowel disorders (49), irritable bowel syndrome (49), gastric bypass surgery (51), metabolic disorders (52), and intestinal inflammation (45, 53, 54), perhaps due to many *Proteobacteria* species having highly immunogenic lipopolysaccharide in the cell wall in comparison to other Gram-negative bacteria (55, 56). From these findings, *Proteobacteria* has been considered to reflect the “unstable structure of the gut microbial community” (45), and the abundance of *Proteobacteria* in this population may be a sign of an imbalance of the gut microbiota or dysbiosis, suggesting gut microbial immaturity compared to children who are healthy.

Further, though this study did not find an association between undernutrition and *Proteobacteria*, poor nutrition status has been linked with higher *Proteobacteria* abundance and other aberrations in gut microbiota (31, 57–59). In a study of 20 children from 0 to 2 years of age living in slums in southern India, healthy children had a higher prevalence of *Bacteroidetes*, Bifidobacterium longum, and Lactobacillus mucosae, compared to stunted children, who harbored more potentially pathogenic organisms such as *Desulfovibrio* and *Campylobacterales* (60). Similarly, in a case study of two children (one healthy, one malnourished) living in an urban slum of Kolkata, India, a higher prevalence of *Campylobacterales* and *Clostridiales* was observed in the malnourished child's gut microbiome (59). Another study in a Bangladesh slum among healthy and malnourished children found a high prevalence of *Klebsiella* and *Escherichia* with a decrease in *Bacteroidetes* and other anaerobes as well as *Lactobacillus* in the malnourished children (57). Similarly, a study in a rural community of West Bengal, India, found a significant clustering of potentially pathogenic groups such as *Escherichia*, *Streptococcus*, and *Shigella* in severely malnourished children compared to healthy children 0 to 60 months of age (61). In the gut microbiomes of Malawian twins discordant for kwashiorkor, gut microbiota were causal in the development of kwashiorkor after performing mechanistic studies in mice; upon examining their gut microbiome, the mice that had developed kwashiorkor had more members of *Proteobacteria*, particularly Bilophila wadsworthia, which caused systemic inflammation in specific-pathogen-free mice (62) as well as Clostridium innocuum, a member of *Firmicutes* and associated with sudden infant death syndrome (63).

### Anthropometry.

The prevalence of poor growth was high in this study, and anthropometric measurements were found to be differentially associated with the gut microbiota, which parallels a recent review (2). In this study, head circumference was positively associated with α-diversity. To our knowledge, studies examining head circumference in relation to the gut microbiome in children have only been done in neonatal and/or premature infants. One study found that administration of a synbiotic, which included members of *Firmicutes* and *Actinobacteria* as well as fructo-oligosaccharides, resulted in a lower odds of head circumference below the 10th percentile, compared to a control group after 1 year of supplementation (64). In another study, certain genera of the maternal microbiota and maternal SDI were positively correlated with neonatal male head circumference (65). In newborn preterm infants, receiving an intervention of 10 to 15 g of medically graded bee honey (a source of oligosaccharides) daily was associated with increased head circumference after 2 weeks, in addition to increased colonization with Bifidobacterium bifidum compared to control receiving no intervention (66). However, to our knowledge, head circumference has not been examined in concert with other nutritional metrics in relation to the gut microbiota diversity or composition in older infants and children.

Increasing weight-for-length Z-score (WLZ) was inversely associated with SDI. When stratified by sex, these associations were present only in male children. Poor growth outcomes in male children compared to female children have been previously observed (67–70). Earlier studies in undernourished infants and their microbiota have found lower microbial diversity with stunting (71), underweight and wasting (57), or severe acute malnutrition (30). Other studies have found no differences in diversity in mice which received microbiota from either severely stunted or nonstunted infants after 30 days postcolonization (72), or in Malawian infant weight-for-age Z-scores (WAZ) between 12 and 18 months of age (73). A potential explanation for our results is our small sample size, or some unmeasured environmental factor(s) may have influenced the association between WLZ and the gut microbiota in this cross-sectional analysis. However, our data suggest that both age and sex should be considered when examining growth and the gut microbiota of infants and children, particularly in lower-resource settings.

### Biomarkers.

In this study, hemoglobin concentration was associated with decreases in Faith’s phylogenetic diversity (Faith’s PD). A previous study of rural Kenyan infants and children found that hemoglobin concentration was not associated with microbial diversity but was positively correlated with numbers of Escherichia coli, a member of the *Proteobacteria* phylum (74, 75). Further, in anemic infants, a positive correlation was found between hemoglobin concentration and *Actinomycetales*, an order of *Actinobacteria* (74, 75). These findings together suggest that further examination of hemoglobin and the gut microbiota would be informative.

### Dietary intakes and feeding practices.

Consumption of fat was consistently associated with α- and β-diversity in this study. Specifically, the log ratio between two genera of the *Firmicutes* phylum, the *Lactococcus/Anaerococcus* log ratio, was found to be significantly higher among children who consumed oils and fats compared to children who did not consume oils and fats the previous day. While these particular genera have not been specifically explored in association with dietary fat intake, a recent meta-analysis of 27 studies done with mice and humans found that high-fat diets reproducibly changed gut microbial community structure, including increased *Firmicutes* relative abundance, but had no consistent association with diversity (76). In children 1 to 6 years of age, European children with fat constituting 44 to 47% of their diet, *Bacteroidetes* and *Firmicutes* were more abundant than in African children whose diets were made up of 25 to 28% fat (77). Some studies of children and infants do suggest an association between fat intake and microbial diversity. In a study in premature infants, supplemental polyunsaturated fatty acids increased bacterial diversity (78), similar to our findings from the Faith’s PD redundancy analysis. Increased fat intake from complementary foods becomes an important source of energy as breast milk consumption decreases (79), particularly in populations at higher risk of undernourishment; our finding of higher fat intake from complementary foods may reflect greater dietary quality, translating to an increase in *Firmicutes* (80) and greater microbial diversity.

Current breastfeeding was also associated with greater α-diversity. Previous studies have found lower α-diversity in breastfed individuals, as breast milk selects for microbiota capable of digesting particular human milk oligosaccharides (HMO) present in breast milk (81–83), such as *Bifidobacterium*, which may suppress the expansion of other microbiota incapable of digesting HMO, resulting in lower diversity (8). In these studies, *Bifidobacterium* spp. dominated the gut microbiota as a result of breast milk consumption, in contrast to our study where *Proteobacteria* represented over 80% of the taxonomic composition despite the report of current breastfeeding in over 80% of participants. We observed higher α-diversity when *Proteobacteria* abundance was relatively low (data not shown), which could allow the expansion of members from other phyla; indeed, we found an inverse association between *Actinobacteria* and Faith’s PD (data not shown). Understanding associations between diet and gut microbiota in populations living in environments with higher risk for undernutrition and poor sanitation may require additional scrutiny compared to populations in higher-income countries.

### Strengths.

There are several strengths of this study. This is the first study to examine the gut microbiota among Indian children living in urban slums of Mumbai, and children from five slums were sampled and analyzed, improving generalizability of findings to children in this age group from other urban slums of Mumbai. We used rectal swabs to sample the microbiota, which has multiple benefits. They are easily obtained and convenient (84). They are stored immediately after sampling, resulting in a lower risk of contamination (84). Short-term storage at room temperature has been shown to have had no impact on composition of gut microbiota (84). They are appropriate for sampling from this age group (85–87). Skin bacterial contamination has been none or low (84). Importantly, the microbiome profile has been shown to be comparable to bulk stool samples in previous studies (48, 84, 85, 87). As another strength, we followed protocols from the Earth Microbiome Project (88, 89), such as DNA extraction and sequencing protocols and 16S rRNA hypervariable region selected, to facilitate interstudy comparability. Further, we analyzed data using state-of-the-art bioinformatics methods (90, 91), including the fragment insertion method to acquire the taxonomic assignments of sequences, which provides advantages over *de novo* phylogenies, including accurate branch lengths, multistudy meta-analyses, and mixed region meta-analyses (36). Use of the V4 hypervariable 16S rRNA region has been shown to be especially appropriate for infant microbiota investigations, as this region tends to allow better recovery of *Bifidobacterium* (92), making our observation of very low bifidobacteria in this population even more stark.

### Limitations.

This study has some limitations. First, this pilot study sample was comprised of the first 53 participants from whom a rectal swab was able to be collected between June and July 2017 (consecutive sampling); as a result, we did not include samples from infants from the full screening period of the parent trial (93): specifically, 5 out of 20 communities sampled are represented in this cross-sectional analysis, which may present selection bias. In comparison to the rest of participants screened, we found that there were no dissimilarities in age, sex, other sociodemographic characteristics, but we did find differences in other measures, including a lower proportion of vaginal births in the microbiome subset (see Table S1 in the supplemental material). The difference in the higher proportion of vaginal births is unexpected, but it may be dependent on the subset of communities included and specific cultural practices. Another limitation in this study is the possibility of error due to the inherent drawbacks of a 24-h dietary recall questionnaire. Recall bias due to the mother’s inability to accurately recall the child’s dietary intake, as well as information bias due to the limitations in food composition databases to convert the reported food consumption to energy and nutrient intakes, may limit the validity of the dietary information collected (94). However, the diet in early childhood is relatively simple with lower dietary diversity than an adult’s diet; in addition, we used the recently updated Indian food composition database (95), suggesting that this is a likely relatively minor weakness of the study. Another limitation is that few children consumed adequate dietary intakes from complementary foods, limiting power for statistical analysis. However, this finding parallels the results from India’s 2016–2018 Comprehensive National Nutrition Survey in children under 2 years of age, suggesting increased generalizability of the cohort (96). Finally, as we conducted 16S rRNA sequencing, we were unable to include an investigation of the functional potential of the gut microbiota, given that algorithms used to predict function, such as Phylogenetic Investigation of Communities by Reconstruction of Unobserved States (PICRUSt) (97), have been validated only on adult populations and therefore are not appropriate for infant populations.

### Conclusions.

This sample of 10- to 18-month-old children living in urban slums of Mumbai, India, had high prevalence of poor growth and nutrient deficiencies, as well as a dominance of *Proteobacteria* in the gut. Anthropometry (head circumference, weight for age), nutritional biomarkers (hemoglobin), and diet (fat intake, iron intake, current breastfeeding) were associated with gut microbiota composition and diversity. Further longitudinal research examining comprehensive nutritional status and the gut microbiota in similar populations is warranted, given that multiple markers of nutrition—growth, biomarkers, diet, and feeding practices—were associated with the gut microbiota.

## MATERIALS AND METHODS

### Study population, setting, and design.

Participants were children between 10 and 18 completed months of age living in urban slums of Mumbai, India (including the eastern wards of Khar, Santacruz, and Bandra) who provided informed caregiver consent to be screened for enrollment into the parent study, a randomized controlled nutrition intervention trial (Clinicaltrials.gov ID: NCT02233764) (93). This exploratory cross-sectional study examined a subset of participants at screening, prior to enrollment (67).

The protocol was reviewed and approved by the Inter Systems Biomedical Ethical Committee (ISBEC) (Mumbai, Maharashtra, India), St. John’s Research Institute (SJRI) Institutional Ethics Committee (IEC), and the Institutional Review Board (IRB) of Cornell University. In addition, permissions to conduct the study were obtained from the Health Ministry Screening Committee of India (Indian Council of Medical Research). Informed consent was obtained from all caregivers in an audio/visual format per Indian Government guidelines (98).

Screening data for this cross-sectional study were collected from June to July 2017; screening data for the parent trial were collected between March and November 2017. Caregivers who had at least one 10- to 18-month-old child as identified during a census survey were invited to come to the study center, the Centre for the Study of Social Change (CSSC) (Bandra East, Mumbai, India), with their child to be screened for eligibility in the randomized trial. Inclusion criteria for enrollment into the parent trial have been described previously (93). The sample size constituted the first 53 stool samples to be collected and next-generation-sequenced and is therefore a convenient sample for this exploratory study. All children were provided 400 mg albendazole as recommended by the World Health Organization (99) during screening under supervision by the study physician.

### Anthropometry.

Trained research assistants collected anthropometric measurements using standardized procedures (100). The average of duplicate (recumbent length, mid-upper-arm circumference, head circumference) measurements was used as the final measurement. The weight of each child was measured using Rice Lake and Seca 703 body weight scales to the nearest 0.01 kg and calculated as the difference in weight of the child’s caregiver alone compared to the weight of the caregiver holding the child, both wearing standard attire (without shoes) (GmbH & Co. KG, Hamburg, Germany). Child recumbent length was measured to the nearest 0.1 cm using an infant length board (ShorrBoard; Weigh and Measure LLC, Olney, MD, USA). Infant anthropometric Z-scores were computed using WHO International Growth References (version 3.2.2, 2011).

### Demographic data, dietary intakes, feeding practices, and health history.

Research assistants collected maternal and child demographic and health history data through interviews with caregivers. These variables included the age of the child, sex of the child, child’s birth weight (low birthweight was defined as less than 2.5 kg [101, 102] and determined by caregiver’s recall and confirmed by maternal/child health card), dietary information, and birth/delivery mode. The child’s health history data were reported to the study physician by the mother, a physical examination was conducted by the study clinician, and morbidity data were recorded as a report of the child having had any occurrence of diarrhea, fever, or cough within the past month. In addition to breastfeeding status information, dietary food group consumption data from the Infant and Young Child Feeding (IYCF) questionnaire were collected (103). These IYCF dietary food groups A to O include the following: A, porridge, bread, rice, noodles, or other foods made from grains; B, pumpkin, carrots, squash, or sweet potatoes that are yellow or orange inside; C, white potatoes, white yams, manioc, cassava, or any other foods made from roots; D, any dark green leafy vegetables; E, ripe mangoes, ripe papayas; F, any other fruits or vegetables; G, liver, kidney, heart, or other organ meats; H, any meat, such as beef, pork, mutton, lamb, goat, chicken, or duck; I, eggs; J, fresh or dried fish, shellfish, or seafood; K, any foods made from beans, peas, lentils, nuts, or seeds; L, cheese, yogurt, paneer, butter, milk, or other milk products; M, any oil, fat, palmolein, butter, or foods made with any of these; N, any sugary foods such as chocolate, sweets, candies, pastries, cakes, or biscuits; and O, condiments for flavor such as chilies, spices, herbs, or fish powder. IYCF food groups B, C, D, E, and F were combined to reflect consumption of all fruits and vegetables in analysis. Dietary intakes of the child were estimated using 24-h dietary recall administered to their mother or caregiver; nutrient intakes were calculated using the updated Indian Food Composition Tables (95) via CS Dietary System software (CS Dietary System, version 1.1). These intakes represent a conservative estimate of macro- and micronutrients consumed from only complementary foods, and no nutrient contributions from breast milk. Dietary intakes were adjusted for energy using multivariate nutrient residual models which included log calories as a constant, i.e., microbiota outcome = b_1_Nutrient residual + b_2_Calories (104).

### Biological specimen collection.

At the study center (CSSC), a pediatric phlebotomist applied topical anesthetic (Prilox Cream [lidocaine with prilocaine]; Neon Laboratories Limited, Mumbai, India) and performed topical antisepsis before collecting blood from the antecubital vein. After centrifugation to separate serum from whole blood, blood was divided into aliquots and immediately transported (within a range of 1 to 6 h after collection) to SRL Diagnostics (Goregaon, Mumbai, India) for immediate analysis as well as storage at −80°C for future batch analyses of nutrition status and immune function. Complete blood counts, including hemoglobin were immediately analyzed (DXH 600 Coulter Counter) (intra-assay coefficient of variation [CV], 0.43%). Serum ferritin was measured using electrochemiluminescence (Cobas8000) (intra-assay CV, 4.5%) (limit of detection [LOD] <0.5 ng/ml). Serum zinc was measured using FAAS with D2 correction (Aanalyst800) (intra-assay CV, 4.99%). C-reactive protein (CRP) was measured using nephelometry (BN II nephelometer) (intra-assay CV, 5.24%).

Stool samples were collected using Copan FecalSwab Regular Flocked Collection kit (Nylon FLOQSwab and tube containing 2 ml Cary-Blair medium) (Thermofisher, catalog no. R723487) by inserting the swab gently 2 to 3 cm into the rectum and rotating 360 degrees until fecal material was visible on the swab. The rectal swab was then stored at 4°C for a maximum of 48 h and subsequently stored at −20°C for a maximum of 1 month until DNA extraction (per the manufacturer’s instructions). Two separate swabs per participant were collected to ensure maximal DNA recovery.

### DNA extraction and 16S rRNA gene next-generation sequencing.

Samples were shipped on ice packs to Genotypic Technology in Bangalore, India, for DNA extraction (modified from the MoBio DNeasy PowerSoil HTP 96 kit Instruction Manual, per instructions for DNA extraction proposed by the Earth Microbiome Project 2018 instructions [105, 106]) and sequencing. The concentration and purity of samples were estimated using the Nanodrop spectrophotometer and Qubit fluorometer. Genomic DNA (25 ng) was amplified for 26 cycles using KAPA HiFi HotStart PCR kit (Kapa Biosystems Inc., Boston, MA, USA). The V3-V4 region was targeted for library construction using 341F/806RB primers modified as described previously (107–110). The forward PCR oligonucleotide (50 bp) contained a 5′ Illumina sequencing adapter, a 10-nucleotide (nt) pad sequence, and the 341 16S specific linker-primer sequence (5′-CCTACGGGNGGCWGCAG-3′). The reverse PCR oligonucleotide (55 bp) contained the 3′ reverse complement of an Illumina sequencing adapter, the 12-nt Golay barcode, a 10-nt pad sequence, and the 16S specific 806R (modified) reverse linker-primer sequence (5′-CCGGACTACNVGGGTWTCTAAT-3′).

The V4 region was chosen to correspond with the reverse primer of the Earth Microbiome Project (EMP), and the V3 region was included in the single fragment to facilitate better alignment during the subsequent analysis. The forward and reverse primer concentrations (0.2 μM each) were analyzed on a 1.2% agarose gel. Round 1 PCR amplicons (1 μl, 1:10) were used for round 2 indexing PCR by amplifying round 1 PCR amplicons for 10 cycles to add Nextera adapters (Nextera XT v2 index kit; Illumina, USA). Round 2 PCR amplicons were analyzed on 1.2% agarose gel. Amplicons were sequenced on Illumina MiSeq, using 275 bp x 2 paired-end sequences by Genotypic Laboratory (Bangalore, India).

### Bioinformatics processing.

Demultiplexing of paired-end reads was performed using bcl2fastq v1.8.4 (111). Further processing was performed using the open-source bioinformatics pipeline, QIIME 2 version 2019.7, installed in a conda environment in Linux CentOS (90). Sequence primers were quality filtered and trimmed using a length of 100 bp via the Deblur workflow, using a minimum read number of 1 and trim length of 100 bp (36, 112, 113). The resulting quality-filtered feature table of sequence variants (equivalent to 100% operational taxonomic units [OTUs]) was visually summarized using the qiime feature-table summarize command to generate descriptive statistics. For phylogenetic diversity analysis, we used the fragment insertion method (114–117) using the Greengenes 13_8 reference database at 99% (command qiime fragment-insertion sepp) as detailed earlier (118). Chloroplast and mitochondrial sequences were filtered out of the resulting table using the qiime taxa filter-table command.

The qiime feature-table rarefy command was used to rarefy the data (by random subsampling) to a sampling depth of 38,012, the deepest sampling depth possible that included all 53 samples (119). The qiime diversity alpha and qiime diversity beta-phylogenetic plug-ins were used to compute α-diversity and β-diversity metrics on the rarefied sequence variant table. Measures of α-diversity analyzed included the Shannon diversity index (SDI) (38), and Faith’s phylogenetic diversity (Faith’s PD) (39). β-Diversity was measured using unweighted and weighted UniFrac to consider the relative abundance of taxa in addition to presence or absence information (120, 121) as well as DEICODE (42).

### Statistical analysis.

We first analyzed continuous variables for normality using the Shapiro-Wilk test. If data were not normally distributed, median and interquartile range (IQR) values were reported. Continuous data were assessed for correlational relationships using Spearman correlation, and medians (IQRs) were compared between groups using the Hodges-Lehmann-Sen test. Categorical data were compared between groups using the chi square test; Fisher’s exact test was used in analyses where at least 25% of expected counts were less than five.

Linear regression was performed to examine the association between exposures of poor nutrition status and gut microbiota outcomes, percent relative abundance, and α-diversity metrics, SDI and Faith’s PD. To identify potentially confounding factors, nutritional exposure variables associated with the outcome of interest at *P* < 0.20 in univariate analysis were included in the multivariate model; only those correlates were retained in the model that were associated with the outcome with a *P* value of ≤0.05 (122). All analyses were adjusted for age and sex.

We also identified nonredundant covariates using a forward stepwise redundancy analysis (RDA) with the *vegan* package in R. This analysis estimates the linear cumulative and independent effect size of each nonredundant covariate on microbiome diversity variation (123). For RDA analysis, after removing colinear variables, we included dietary, sociodemographic, and clinical correlates in the model.

β-Diversity (unweighted and weighted UniFrac metrics) was visualized by principal coordinate analysis (PCoA) using the Emperor software through QIIME 2, using abundance data to determine clustering patterns between the participants (124). To determine whether UniFrac distances clustered among participants with particular characteristics, we used the qiime diversity beta-group-significance command to run a permutational multivariate analysis of variance (PERMANOVA) (40, 125) test to determine whether (weighted or unweighted) UniFrac distances between participants within a group were more similar to each other than they were to participants representing the other (reference) group. To test for homogeneity of multivariate dispersions, PERMDISP (41) was run to compare within-group spread among groups using the average distance between individual observations to their group centroid to identify the relative spreads of data cloud shapes among groups (126).

To address sparsity in our data, we also examined which taxa strongly influenced clustering using default parameters of the robust Aitchison principal component analysis (RPCA) DEICODE (version 0.2.4) QIIME 2 plug-in (qiime deicode rpca) (42). DEICODE is robust to high levels of sparsity, such that zero values do not influence the resulting ordination. Any taxon identified to the species level was considered misidentified, as 16S rRNA sequencing is susceptible to species-level misidentifications. The resulting compositional biplots (127) were visualized in QIIME 2 using Emperor to assess the 25 features with the highest magnitudes, i.e., those expected to be important in causing separation in the data set (https://forum.qiime2.org/t/how-to-make-pcoa-biplot-in-r-using-q2-deicode-ordination/8377/6) (--p-number-of-features, 25). We performed PERMANOVA on the DEICODE results.

The feature loadings in a compositional biplot produced by DEICODE output were then visualized in the Qurro version 0.4.0 QIIME 2 plug-in (command qiime qurro loading-plot) to display a plot of feature loadings for a given axis in the biplot alongside a plot of the log ratios of selected features’ abundances within samples (43). The rank plot field was unadjusted, and therefore, the feature loadings from axis 1 of the biplot were assessed. The log ratios of taxa at the extremes of the Qurro rank plot were selected to compare (using Student’s *t* test assuming unequal variances and no multiple comparisons correction applied) between group characteristics that were statistically significantly different from the DEICODE results.

Exposures of interest included anthropometric indices, including birth weight, current weight, mid-upper arm circumference, length, head circumference, weight-for-age Z-score (WAZ), length-for-age Z-score (LAZ), and weight-for-length Z-score (WHZ) (128); blood biomarkers, including ferritin, zinc, and C-reactive protein, hemoglobin concentration; report of diarrhea, cough, or fever within the past 4 weeks; and dietary and feeding practices, including dietary intakes of macro- and micronutrients, current breastfeeding, IYCF indicators (103); as well as data on birth mode (vaginal or Caesarean). All analyses were adjusted for age and sex where appropriate.

Selection bias was examined by comparing characteristics (such as age, sex, and other clinical and dietary characteristics) of the sample in this cross-sectional study with the rest of the screened participants. All exposure/outcome combinations were tested and are reported in supplemental tables; we reported those of interest and those that were statistically significant.

After statistical tests, we utilized false discovery rates (FDR) per the Benjamini and Hochberg approach (37), as *post hoc* multiple testing corrections. All analyses were two sided, and differences between groups were considered significant at *P* < 0.05. Data were analyzed using SAS version 9.4 (SAS Institute, Cary, NC, USA), R Studio (R Foundation), and QIIME 2 version 2019.7.

### Data availability.

These data were subject to cross-checking and confirmation by the Cornell Institute for Social and Economic Research (CISER) (member of DataCite, https://doi.org/10.5281/zenodo.556235) to ensure reproducibility; data sets and code are available upon request at https://doi.org/10.6077/zrvc-pc31. The data that support the findings of this study are openly available in NCBI BioProject at https://www.ncbi.nlm.nih.gov/bioproject/PRJNA657036. The DNA sequences corresponding to the 16S rRNA gene data in this study have been submitted as raw fastq files to the SRA at https://www.ncbi.nlm.nih.gov/sra/PRJNA657036. Various QIIME 2 files, including Qurro plots, are available at https://github.com/knightlab-analyses/nutrition-gut-microbiota-mumbai.
