# Supplementary material for: Nutrition and the Gut Microbiota in 10- to 18-Month-Old Children Living in Urban Slums of Mumbai, India
Source: mSphere. 2020 Sep 23;5(5):e00731-20. doi: 10.1128/mSphere.00731-20 (PMC7568645; doi:10.1128/mSphere.00731-20)
Supplement: TABLE S1 [file mSphere.00731-20-st001.docx]

| **Table S1. Comparison of microbiome subset with excluded screening population** | | | | | |
| --- | --- | --- | --- | --- | --- |
| **Characteristics** | **n** | **Microbiome Subset** | **n** | **Screening Population** | ***P-*value^a^** |
|  |  | **Median (IQR) or**  **n/N (%)** |  | **Median (IQR) or**  **n/N (%)** |  |
| Vaginally delivered (vs Caesarean) | 51 | 29 (56.9) | 269 | 198 (73.6) | 0.02 |
| Mid-upper arm circumference (cm) | 53 | 14.8 (14.1, 15.1) | 271 | 14.2 (13.5, 14.8) | 0.0001 |
| Calories (kcal)^b^ | 52 | 393.0 (270.0, 645.5) | 312 | 550.5 (308.5, 805.5) | 0.04 |
| Protein (g)^b^ | 52 | 13.6 (8.1, 21.6) | 312 | 18.0 (10.4, 27.3) | 0.03 |
| Fat (g)^b^ | 52 | 12.9 (8.9, 20.8) | 312 | 20.0 (9.0, 30.1) | 0.01 |
| Cough today or within past 4 weeks | 51 | 7 (13.7) | 248 | 88 (35.5) | 0.002 |
| Consumed orange root vegetables yesterday^c^ | 51 | 9 (17.6) | 265 | 22 (8.3) | 0.04 |
| Consumed any fruits and vegetables yesterday^d^ | 50 | 13 (26.0) | 264 | 115 (43.6) | 0.02 |
| Consumed dairy yesterday^e^ | 50 | 26 (52.0) | 262 | 181 (69.1) | 0.02 |
| Consumed sugary foods yesterday^f^ | 51 | 30 (58.8) | 265 | 208 (78.5) | 0.003 |
| Consumed condiments yesterday^g^ | 50 | 6 (12.0) | 263 | 70 (26.6) | 0.03 |
| ^a^P-values generated using Hodges-Lehmann Sen test or Chi Square test for continuous or categorical variables, respectively.  ^b^Absolute (unadjusted) dietary intakes.  ^c^Infant and young child feeding [IYCF, (World Health Organization)] Food Group B.  ^d^IYCF Food Group F.  ^e^IYCF Food Group L.  ^f^IYCF Food Group N.  ^g^IYCF Food Group O**.** | | | | | |
|  |  |  |  |  |  |
|  |  |  |  |  |  |
|  |  |  |  |  |  |
