# Supplementary figures and images for: Nutrition and the Gut Microbiota in 10- to 18-Month-Old Children Living in Urban Slums of Mumbai, India
Source: mSphere. 2020 Sep 23;5(5):e00731-20. doi: 10.1128/mSphere.00731-20 (PMC7568645; doi:10.1128/mSphere.00731-20)

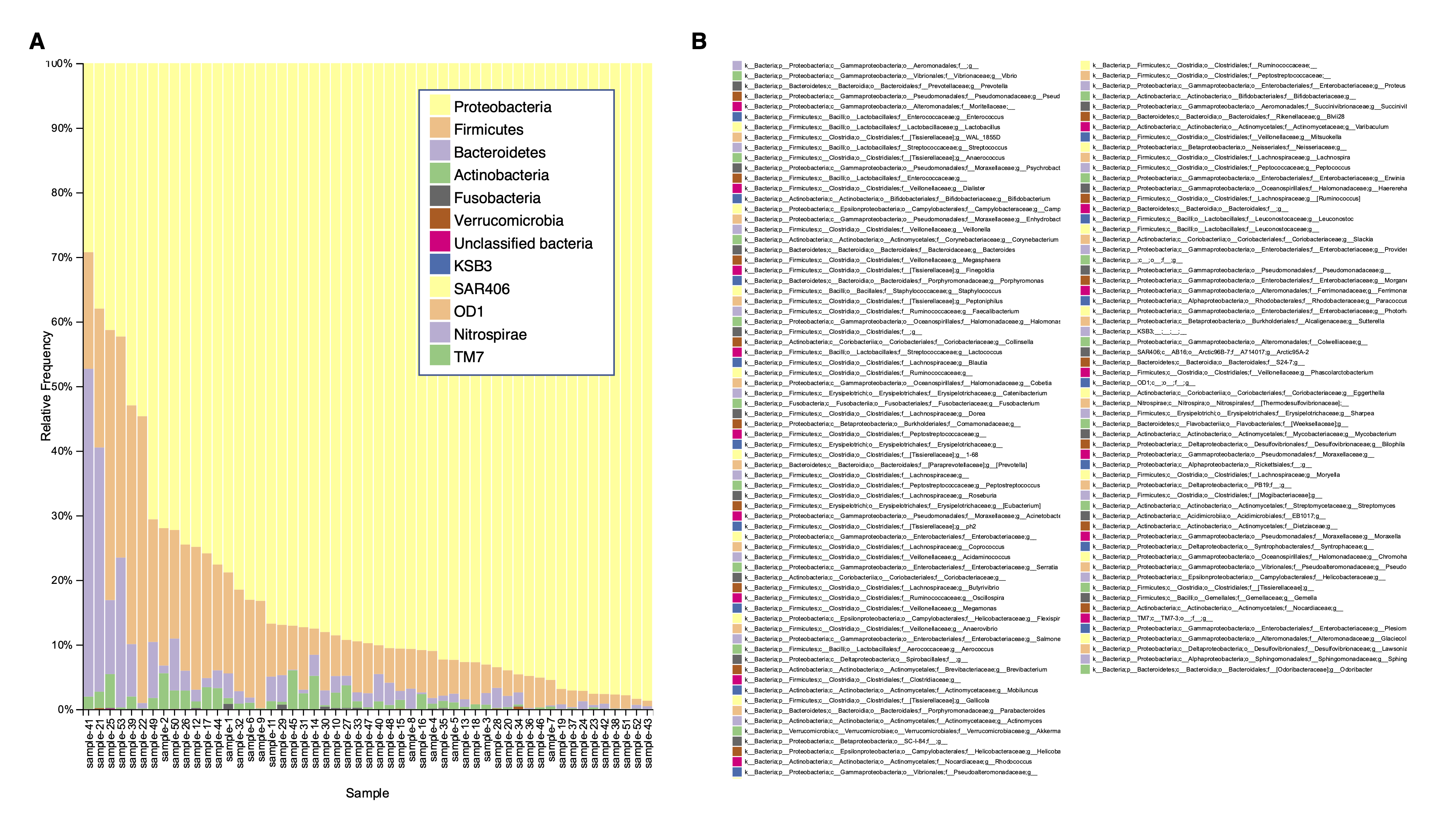

Supplement: FIG S1 [file mSphere.00731-20-sf001.tif]

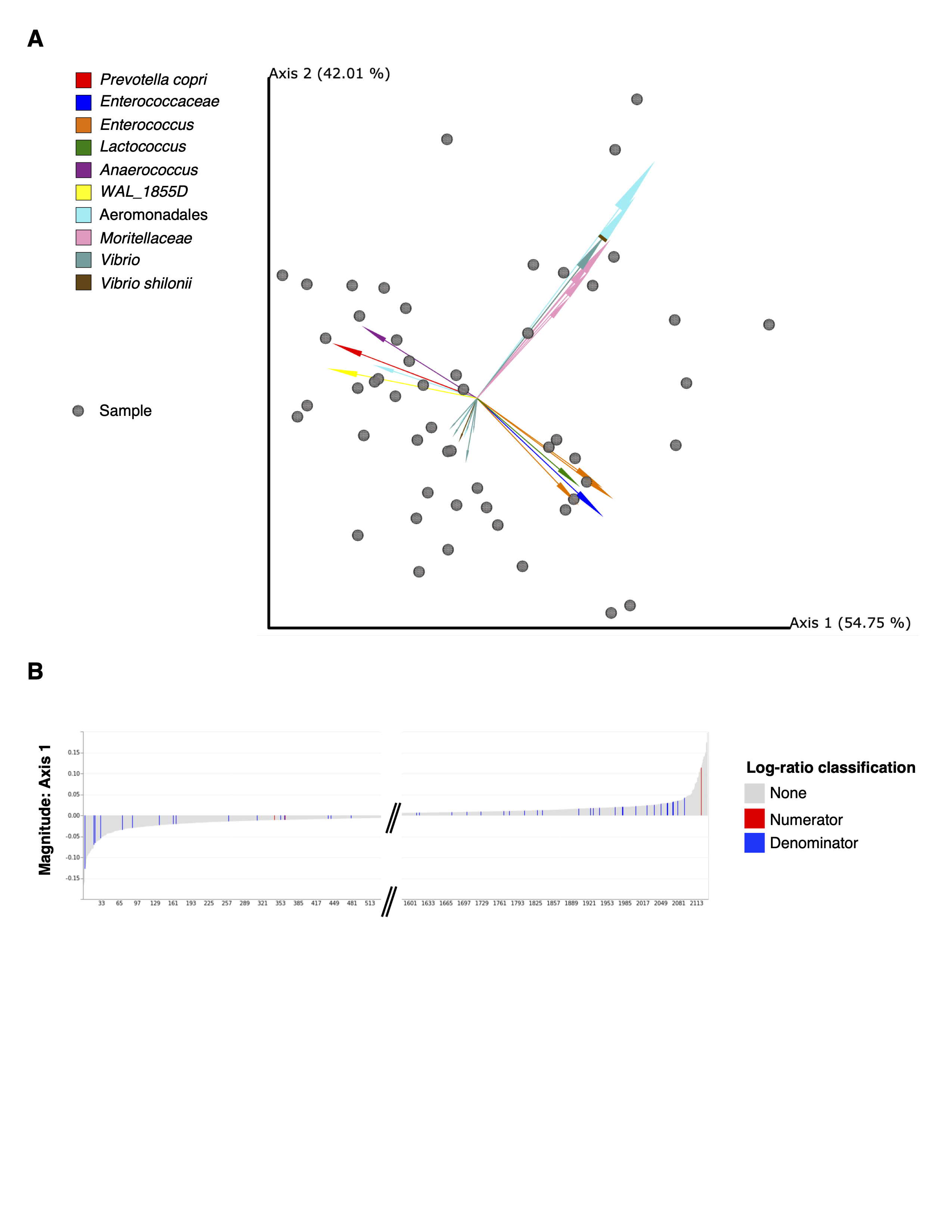

Supplement: FIG S2 [file mSphere.00731-20-sf002.tiff]
