# Supplementary material for: Nutrition and the Gut Microbiota in 10- to 18-Month-Old Children Living in Urban Slums of Mumbai, India
Source: mSphere. 2020 Sep 23;5(5):e00731-20. doi: 10.1128/mSphere.00731-20 (PMC7568645; doi:10.1128/mSphere.00731-20)
Supplement: TABLE S2 [file mSphere.00731-20-st002.docx]

| **Table S2. Correlates of phylum relative abundance: all tests^a^** | | | | | |
| --- | --- | --- | --- | --- | --- |
| **Characteristic** | | **Actinobacteria (%)** | **Bacteroidetes (%)** | **Firmicutes (%)** | **Proteobacteria (%)** |
| **Demographic & maternal** | **n** | **β (%) (95% CI)** | **β (%) (95% CI)** | **β (%) (95% CI)** | **β (%) (95% CI)** |
| Age (months) | 53 | **0.20 (0.01, 0.38)^i^** | 0.58 (-0.50, 1.66) | **1.16 (-0.07, 2.38)^j^** | **-1.93 (-3.90, 0.04)^j^** |
| Female | 53 | -0.24 (-1.07, 0.59) | 3.06 (-1.65, 7.77) | 2.72 (-2.76, 8.20) | -5.46 (-14.24, 3.32) |
| Vaginally delivered (vs Caesarean) | 51 | -0.29 (-1.13, 0.54) | -2.60 (-7.37, 2.17) | 0.27 (-5.19, 5.73) | 2.64 (-6.04, 11.31) |
| **Anthropometry** | **n** | **β (%) (95% CI)** | **β (%) (95% CI)** | **β (%) (95% CI)** | **β (%) (95% CI)** |
| Birth weight (kg) | 53 | -0.40 (-1.10, 0.30) | -1.50 (-5.40, 2.50) | **1.60 (-3.00, 6.10)^j^** | 0.30 (-7.00, 7.60) |
| Weight (kg) | 51 | 0.03 (-0.30, 0.36) | **-1.49 (-3.39, 0.40)^j^** | **-1.96 (-4.09, 0.18)^j^** | **3.43 (0.05, 6.82)^i^** |
| Length (cm) | 51 | -0.008 (-0.14, 0.12) | -0.31 (-1.07, 0.45) | **-0.58 (-1.43, 0.27)^j^** | **0.90 (-0.46, 2.26)^j^** |
| Mid-upper arm circumference (cm) | 53 | 0.14 (-0.33, 0.61) | **-2.63 (-5.29, 0.02)^j^** | **-2.22 (-5.28, 0.84)^j^** | **4.76 (-0.05, 9.57)^j^** |
| Head circumference (cm) | 52 | **0.30 (0.02, 0.57)^i^** | -0.11 (-1.76, 1.54) | -0.22 (-2.09, 1.65) | 0.03 (-2.96, 3.02) |
| Head circumference-for-age Z-score (HCZ)^a^ | 52 | **0.35 (-0.03, 0.74)^j^** | -0.38 (-2.65, 1.90) | -0.75 (-3.38, 1.87) | 0.77 (-3.47, 5.02) |
| Length-for-age Z-score (LAZ)^a^ | 51 | -0.03 (-0.38, 0.32) | -1.06 (-3.04, 0.93) | **-1.86 (-4.12, 0.41)^j^** | **2.94 (-0.73, 6.60)^j^** |
| Weight-for-age Z-score (WAZ)^a^ | 52 | -0.006 (-0.36, 0.34) | **-1.84 (-3.78, 0.10)^j^** | **-2.51 (-4.72, -0.29)^i^** | **4.36 (0.82, 7.90)^i^** |
| Weight-for-length Z-score (WLZ)^a^ | 51 | 0.05 (-0.36, 0.45) | **-2.26 (-4.50, -0.02)^i^** | **-3.10 (-5.65, -0.56)^i^** | **5.33 (1.25, 9.41)^i^** |
| **Biomarkers** | **n** | **β (%) (95% CI)** | **β (%) (95% CI)** | **β (%) (95% CI)** | **β (%) (95% CI)** |
| Serum ferritin (ng/mL) | 44 | -0.0007 (-0.01, 0.01) | -0.04 (-0.12, 0.05) | -0.02 (-0.11, 0.07) | 0.05 (-0.09, 0.20) |
| Serum zinc (µmol/L) | 38 | -0.10 (-0.29, 0.08) | **1.41 (0.21, 2.61)^i^** | -0.31 (-1.60, 0.98) | -0.99 (-3.15, 1.17) |
| CRP >5 mg/L | 39 | **-1.39 (-3.03, 0.25)^j^** | -5.40 (-17.21, 6.40) | 5.96 (-4.41, 16.33) | 0.89 (-17.88, 19.66) |
| Hemoglobin (g/dL) | 43 | **0.28 (-0.08, 0.64)^j^** | -1.13 (-3.26, 1.00) | -0.51 (-2.45, 1.42) | 1.39 (-2.22, 5.00) |
| **Clinical** | **n** | **β (%) (95% CI)** | **β (%) (95% CI)** | **β (%) (95% CI)** | **β (%) (95% CI)** |
| Diarrhea within past month | 51 | -0.42 (-1.52, 0.69) | -2.12 (-8.50, 4.27) | 1.85 (-5.41, 9.10) | 0.65 (-10.94, 12.24) |
| Fever within past month | 51 | -0.43 (-1.32, 0.46) | -3.05 (-8.18, 2.08) | 0.83 (-5.07, 6.73) | 2.63 (-6.75, 12.01) |
| Cough within past month | 51 | 0.17 (-1.05, 1.39) | -2.27 (-9.31, 4.78) | 2.65 (-5.34, 10.64) | -0.46 (-13.26, 12.33) |
| **Dietary intakes^b^** | **n** | **β (%) (95% CI)** | **β (%) (95% CI)** | **β (%) (95% CI)** | **β (%) (95% CI)** |
| Calories (kcal) | 52 | -0.0004 (-0.002, 0.001) | 0.004 (-0.004, 0.01) | -0.004 (-0.01, 0.006) | 0.0002 (-0.01, 0.02) |
| Protein (g) | 52 | 0.41 (-1.12, 1.93) | -0.79 (-9.66, 8.09) | 6.28 (-3.63, 16.19) | -5.84 (-21.89, 10.22) |
| Fat (g) | 52 | -0.16 (-0.88, 0.56) | 0.42 (-3.77, 4.60) | -2.69 (-7.38, 1.99) | 2.39 (-5.19, 9.98) |
| Monounsaturated fat (g) | 51 | -0.10 (-0.38, 0.18) | **-1.17 (-2.76, 0.42)^j^** | -0.74 (-2.56, 1.07) | **2.03 (-0.85, 4.91)^j^** |
| Polyunsaturated fat (g) | 51 | **-0.38 (-1.00, 0.25)^j^** | -1.61 (-5.29, 2.07) | -0.38 (-4.56, 3.80) | 2.38 (-4.31, 9.06) |
| Saturated fat (g) | 51 | -0.07 (-0.34, 0.20) | -0.89 (-2.44, 0.67) | -0.49 (-2.26, 1.28) | 1.46 (-1.36, 4.29) |
| Carbohydrate (g) | 52 | 0.10 (-1.70, 1.90) | 0.29 (-10.20, 10.78) | 4.13 (-7.69, 15.96) | -4.46 (23.48, 14.57) |
| Fiber (g) | 52 | **-0.56 (-1.13, 0.0003)^j^** | 0.004 (-4.75, 4.75) | **4.24 (-0.002, 8.48)^j^** | -3.70 (-11.06, 3.67) |
| Calcium (mg) | 52 | 0.18 (-0.22, 0.57) | -1.33 (-3.64, 0.97) | 1.01 (-1.60, 3.63) | 0.19 (-4.05, 4.42) |
| Iron (mg) | 52 | 0.34 (-0.54, 1.23) | -0.70 (-5.92, 4.53) | **7.50 (1.98, 13.03)^i^** | **-7.08 (-16.37, 2.21)^j^** |
| Zinc (mg) | 52 | **-1.13 (-2.09, -0.17)^i^** | -2.86 (-8.79, 3.07) | -2.13 (-8.69, 4.43) | 6.17 (-4.37, 16.72) |
| Vitamin A (µg RAE) | 52 | -0.16 (-0.84, 0.52) | 1.09 (-1.73, 3.91) | -1.28 (-5.80, 3.23) | 0.37 (-6.23, 6.97) |
| **Feeding practices** | **n** | **β (%) (95% CI)** | **β (%) (95% CI)** | **β (%) (95% CI)** | **β (%) (95% CI)** |
| Breastfed yesterday (current breastfeeding) | 48 | -0.004 (-1.13, 1.13) | 2.20 (-4.80, 9.21) | 4.68 (-3.10, 12.46) | -6.87 (-19.34, 5.60) |
| Exclusive breastfeeding duration (months) | 51 | 0.01 (-0.27, 0.30) | -0.19 (-1.82, 1.44) | **1.34 (-0.47, 3.15)^j^** | -1.18 (-4.10, 1.74) |
| Exclusively breastfed >6 months | 51 | -0.19 (-1.06, 0.67) | 0.97 (-4.04, 5.97) | 0.84 (-4.83, 6.51) | -1.61 (-10.64, 7.42) |
| Consumed grains (bread, rice, noodles, porridge) yesterday^c^ | 51 | -0.06 (-1.62, 1.50) | 0.30 (-8.73, 9.32) | 3.17 (-6.99, 13.32) | -3.35 (-19.58, 12.87) |
| Consumed any fruits and vegetables yesterday^d^ | 51 | -0.34 (-1.19, 0.50) | -2.38 (-7.27, 2.51) | **-4.78 (-10.20, 0.64)^j^** | **7.57 (-1.07, -16.22)^j^** |
| Consumed beans, peas, lentils, nuts seeds yesterday^e^ | 51 | 0.29 (-0.55, 1.13) | -2.55 (-7.38, 2.28) | **4.09 (-1.30, 9.49)^j^** | -1.91 (-10.68, 6.87) |
| Consumed dairy yesterday^f^ | 50 | -0.53 (-1.35, 0.28) | 0.78 (-4.16, 5.72) | -0.06 (-5.53, 5.41) | -0.23 (-8.98, 8.52) |
| Consumed oil or butter yesterday^g^ | 51 | 0.10 (-0.86, 1.06) | 0.85 (-4.70, 6.40) | -0.34 (-6.61, 5.93) | -0.58 -10.58, 9.42) |
| Consumed sugary foods yesterday^h^ | 51 | 0.47 (0.43, 1.36) | -1.44 (-6.66, 3.79) | -0.72 (-6.64, 5.19) | 1.79 (-7.64, 11.21) |
| ^a^Analyses adjusted for age and sex (except: age is not adjusted for sex; sex is not adjusted for age; and HCZ, LAZ, WAZ, WLZ are not adjusted for age and sex as calculating these metrics are incorporates age and sex). Analyses not corrected for multiple comparisons. Bolded estimates highlight associations at the p<0.20 or p<0.05 level.  ^b^All intakes adjusted for energy using the nutrient residual method, except for calories.  ^c^Infant and Young Child Feeding (IYCF, World Health Organization) Food Group A.  ^d^IYCF Food groups B–F combined (orange and starchy root vegetables, dark leafy green vegetables, ripe mangoes or papayas, any other fruits and vegetables).  ^e^IYCF Food Group K.  ^f^IYCF Food Group L.  ^g^IYCF Food Group M.  ^h^IYCF Food Group N.  ^i^Association at p<0.05.  ^j^Association at p<0.20. | | | | | |
|  |  |  |  |  |  |
