# Supplementary material for: Nutrition and the Gut Microbiota in 10- to 18-Month-Old Children Living in Urban Slums of Mumbai, India
Source: mSphere. 2020 Sep 23;5(5):e00731-20. doi: 10.1128/mSphere.00731-20 (PMC7568645; doi:10.1128/mSphere.00731-20)
Supplement: TABLE S4 [file mSphere.00731-20-st004.docx]

| **Table S4. Redundancy analysis for α-diversity** | | | | | | |
| --- | --- | --- | --- | --- | --- | --- |
| **Faith's Phylogenetic Diversity** | | | | | | |
| ***Explanatory variable*** | ***R^2^ adj*** | ***Df*** | ***AIC*** | ***F*** | ***p-value*** | ***Effect size*** |
| Age (months) | 0.27 | 1 | 71.93 | 15.96 | 0.002 | 0.27 |
| Iron (mg) | 0.42 | 1 | 62.98 | 11.62 | 0.004 | 0.15 |
| Poly-unsaturated fatty acids (g) | 0.47 | 1 | 59.87 | 4.91 | 0.036 | 0.05 |
| Model initially included non-dietary covariates to determine contribution of all covariates on variation in α-diversity. Dietary intakes are nutrient residuals adjusted for energy. | | | | | | |
|  |  |  |  |  |  |  |
