# Supplementary material for: Nutrition and the Gut Microbiota in 10- to 18-Month-Old Children Living in Urban Slums of Mumbai, India
Source: mSphere. 2020 Sep 23;5(5):e00731-20. doi: 10.1128/mSphere.00731-20 (PMC7568645; doi:10.1128/mSphere.00731-20)
Supplement: TABLE S5 [file mSphere.00731-20-st005.docx]

| **Table S5. 25 highest-magnitude features in the DEICODE biplot** | | | |
| --- | --- | --- | --- |
| **Taxonomic Classifications** | | | |
| k__Bacteria; p__Firmicutes; c__Bacilli; o__Lactobacillales; f__Enterococcaceae; g__Enterococcus; s__ | | | |
| k__Bacteria; p__Firmicutes; c__Bacilli; o__Lactobacillales; f__Enterococcaceae; g__; s__ | | | |
| k__Bacteria; p__Firmicutes; c__Bacilli; o__Lactobacillales; f__Streptococcaceae; g__Lactococcus; s__ | | | |
| k__Bacteria; p__Firmicutes; c__Clostridia; o__Clostridiales; f__[Tissierellaceae]; g__Anaerococcus; s__ | | | |
| k__Bacteria; p__Firmicutes; c__Clostridia; o__Clostridiales; f__[Tissierellaceae]; g__WAL_1855D; s__ | | | |
| k__Bacteria; p__Proteobacteria; c__Gammaproteobacteria; o__Aeromonadales; f__; g__; s__ | | | |
| k__Bacteria; p__Proteobacteria; c__Gammaproteobacteria; o__Alteromonadales; f__Moritellaceae | | | |
| k__Bacteria; p__Proteobacteria; c__Gammaproteobacteria; o__Vibrionales; f__Vibrionaceae; g__Vibrio; s__ | | | |
| k__Bacteria; p__Proteobacteria; c__Gammaproteobacteria; o__Vibrionales; f__Vibrionaceae; g__Vibrio; s__shilonii*^a^* | | | |
| k__Bacteria; p__Bacteroidetes; c__Bacteroidia; o__Bacteroidales; f__Prevotellaceae; g__Prevotella; s__copri*^a^* | | | |
| **Cluster** | **Feature (Sequence) IDs** | **Color** | **Taxonomic classification** *(highest specificity)* |
| Cluster 1 (pink, cyan, brown, teal): | 2d799af3afb804d7ad5a592f7dc494b5 | **brown** | Species; *Vibrio shilonii^a^* |
|  | 3b8c08b4986a3799fbda4de6e0c894b5 | **pink** | Family; *Moritellaceae* |
|  | 1f998a0a932d18dbdcd7f6101c3503c6 | **pink** | Family; *Moritellaceae* |
|  | 595c022b1a3e89d59f0cf40f5e1fe8d1 | **pink** | Family; *Moritellaceae* |
|  | fde4a7bec27aba04642daf4efca972b9 | **pink** | Family; *Moritellaceae* |
|  | ba90f28227843cb45aa495997bf5435f | **cyan** | Order; Aeromonadales |
|  | 9a030044060c6e14e52bdde0ba03ec64 | **cyan** | Order; Aeromonadales |
|  | 75acba4e8b01fcad32d9c8cd86b62466 | **teal** | Family; Vibrionaceae |
| Cluster 2 (orange, green, blue): | 151cbc35848a0232b2bd610c9b23cfcb | **orange** | Genus; *Enterococcus* |
|  | 4cefce8f985a27fac1fc5f4129370840 | **orange** | Genus; *Enterococcus* |
|  | 582040032c9528f269093d5741972244 | **orange** | Genus; *Enterococcus* |
|  | 4cefce8f985a27fac1fc5f4129370840 | **green** | Genus; Lactococcus |
|  | daf32a03a92e665c9b44a483ca65d38e | **blue** | Family; *Enterococcaceae* |
| Cluster 3 (brown, cyan, teal) | b822da2f0ee5a779ee6729a69f5622fa | **cyan** | Order; Aeromonadales |
|  | c0464c6ab19f154fd410d92e66962bc4 | **cyan** | Order; Aeromonadales |
|  | cea2b8fad79ccbc9c18221655b8bf921 | **brown** | Species; *Vibrio shilonii^a^* |
|  | 5a1ce1578ec9d32a2669d12c0b903653 | **teal** | Family; Vibrionaceae |
|  | fd5d13e44a4810e138fea5b47b7fe456 | **teal** | Family; Vibrionaceae |
|  | 9359f433e2ff974f505f3bba7d4a3da3 | **teal** | Family; Vibrionaceae |
|  | 17743415814d069e54f85d4a2e5613bf | **teal** | Family; Vibrionaceae |
|  | f03b00694a79fbe9171067afadf893bd | **teal** | Family; Vibrionaceae |
| Cluster 4 (cyan, purple, red, yellow) | 814609da8ac86c97569053800e8c1040 | **cyan** | Order; Aeromonadales |
|  | 68721cda4a4a5df3340d505b4acd58a9 | **purple** | Genus; *Anaerococcus* |
|  | 22a75c65c45357f8c98a26554b9650ce | **red** | Species; *Prevotella copri^a^* |
|  | ee83961408b0e2988052e8ecf6a9869c | **yellow** | Genus; WAL_1855D |
| ^a^Species identifications may be misclassified due to the resolution inherent to 16S rRNA marker gene analyses (see ***Figure 3, Figure S2***). | | | |
