# Supplementary material for: Nutrition and the Gut Microbiota in 10- to 18-Month-Old Children Living in Urban Slums of Mumbai, India
Source: mSphere. 2020 Sep 23;5(5):e00731-20. doi: 10.1128/mSphere.00731-20 (PMC7568645; doi:10.1128/mSphere.00731-20)
Supplement: TABLE S6 [file mSphere.00731-20-st006.docx]

| **Table S6. Correlates of β-diversity: all tests** | | | | | | | | | | | | | | | |
| --- | --- | --- | --- | --- | --- | --- | --- | --- | --- | --- | --- | --- | --- | --- | --- |
| **Characteristic** | **PERMANOVA^a^** | | | | | | **PERMDISP^b^** | | | | | | **PERMANOVA^a^** | | |
|  | **Unweighted UniFrac** | | | **Weighted UniFrac** | | | **Unweighted UniFrac** | | | **Weighted UniFrac** | | | **DEICODE^c^** | | |
|  | *Test statistic* | *p-value* | *q-value^d^* | *Test statistic* | *p-value* | *q-value^d^* | *Test statistic* | *p-value* | *q-value^d^* | *Test statistic* | *p-value* | *q-value^d^* | *Test statistic* | *p-value* | *q-value^d^* |
| Male (vs female) | 1.27 | 0.08 | 0.09 | 0.92 | 0.38 | 0.38 | 0.05 | 0.80 | 0.82 | 1.71 | 0.22 | 0.19 | 1.12 | 0.33 | 0.33 |
| Vaginal birth (vs Caesarean) | 0.85 | 0.80 | 0.82 | 0.70 | 0.50 | 0.53 | 0.31 | 0.61 | 0.58 | 0.25 | 0.61 | 0.64 | 0.72 | 0.47 | 0.50 |
| Diarrhea (vs no diarrhea) | 1.13 | 0.21 | 0.20 | 0.99 | 0.34 | 0.35 | 3.15 | 0.33 | 0.36 | 0.22 | 0.65 | 0.65 | 0.18 | 0.86 | 0.86 |
| Fever (vs no fever) | 0.92 | 0.63 | 0.64 | 1.66 | 0.15 | 0.17 | 0.32 | 0.59 | 0.61 | 0.65 | 0.45 | 0.45 | 0.19 | 0.83 | 0.85 |
| Cough (vs no cough) | 0.90 | 0.68 | 0.69 | 0.84 | 0.40 | 0.42 | 2.13 | 0.58 | 0.57 | 0.05 | 0.84 | 0.86 | 0.20 | 0.83 | 0.83 |
| Exclusively breastfed > 6 months (vs. <6 months) | 1.05 | 0.31 | 0.32 | 0.45 | 0.73 | 0.75 | 0.31 | 0.62 | 0.63 | 0.06 | 0.82 | 0.81 | 0.04 | 0.98 | 0.97 |
| Current breastfeeding (vs not) | 1.20 | 0.13 | 0.11 | 1.46 | 0.15 | 0.17 | 7.55 | 0.09 | 0.09 | 0.95 | 0.37 | 0.36 | 1.02 | 0.38 | 0.37 |
| Consumed grains (bread, rice, noodles, porridge) yesterday^e^ | 0.94 | 0.57 | 0.56 | 0.23 | 0.94 | 0.94 | 11.23 | 0.21 | 0.22 | 0.01 | 0.95 | 0.97 | 0.73 | 0.50 | 0.48 |
| Consumed any fruits and vegetables yesterday^f^ | 0.83 | 0.87 | 0.84 | 1.63 | 0.16 | 0.16 | 0.001 | 0.97 | 0.96 | 2.75 | 0.12 | 0.13 | 0.98 | 0.37 | 0.38 |
| Consumed beans, peas, lentils, nuts seeds yesterday^g^ | 0.97 | 0.49 | 0.52 | 0.70 | 0.52 | 0.53 | 0.65 | 0.43 | 0.42 | 0.07 | 0.83 | 0.80 | 1.56 | 0.22 | 0.22 |
| Consumed dairy yesterday^h^ | 1.01 | 0.41 | 0.4 | 0.30 | 0.90 | 0.93 | 0.14 | 0.69 | 0.72 | 0.18 | 0.68 | 0.68 | 0.51 | 0.61 | 0.63 |
| Consumed oil or butter yesterday^i^ | 1.39 | 0.05 | 0.04 | 0.81 | 0.45 | 0.44 | 0.19 | 0.75 | 0.77 | 0.53 | 0.50 | 0.47 | 3.54 | **0.04** | **0.04** |
| Consumed sugary foods yesterday^j^ | 1.1 | 0.26 | 0.24 | 0.43 | 0.77 | 0.77 | 0.23 | 0.61 | 0.62 | 0.39 | 0.56 | 0.54 | 0.18 | 0.84 | 0.88 |
| ^a^PERMANOVA, permutational multivariate analysis of variance.  ^b^PERMDISP, test for homogeneity of multivariate dispersions.  ^c^DEICODE, Robust Aitchison Principle Components Analysis (RPCA) to determine which taxa strongly influence clustering.  ^d^Q-values derived from pairwise testing and represent the false discovery rate (FDR) analog of a P-value. Bolded p-values highlight statistical significance (p<0.05).  ^e^Infant and young child feeding (IYCF, World Health Organization) Food Group A.  ^f^Food groups B–F combined (orange and starchy root vegetables, dark leafy green vegetables, ripe mangoes or papayas, any other fruits and vegetables).  ^g^Food Group K.  ^h^Food Group L.  ^i^Food Group M.  ^j^Food Group N. | | | | | | | | | | | | | | | |
|  |  |  |  |  |  |  |  |  |  |  |  |  |  |  |  |
